# Optical changes and association with axial elongation in children wearing orthokeratology lenses of different back optic zone diameter

**DOI:** 10.1186/s40662-023-00344-3

**Published:** 2023-07-01

**Authors:** Biyue Guo, Pauline Cho, Sin Wan Cheung, Randy Kojima, Stephen Vincent

**Affiliations:** 1 Centre for Myopia Research, Optometry Research Clinic, School of Optometry, The Hong Kong Polytechnic University, A136-137, Hung Hum, Kowloon, Hong Kong SAR China; 2 College of Optometry, Pacific University, Oregon, USA; 3 Contact Lens and Visual Optics Laboratory, School of Optometry and Vision Science, Centre for Vision and Eye Research, Queensland University of Technology, Brisbane, QLD Australia

**Keywords:** Orthokeratology, Myopia control, BOZD, Treatment zone, Higher-order aberrations

## Abstract

**Purpose:**

To compare changes in ocular aberrations in children wearing orthokeratology (ortho-k) lenses with a back optic zone diameter (BOZD) of 6 mm (6-MM group) or 5 mm (5-MM group) and their associations with axial elongation (AE) over two years.

**Methods:**

Seventy Chinese children, aged 6 to < 11 years, with myopia between − 4.00 to − 0.75 D, were randomly allocated to 5-MM and 6-MM groups. Ocular aberrations were measured, rescaled to a 4-mm pupil, and fitted with a 6th order Zernike expansion. Measurements, including axial length, were taken prior to commencing ortho-k treatment and then every six months over two years.

**Results:**

After two years, the 5-MM group displayed a smaller horizontal treatment zone (TZ) diameter (by 1.14 ± 0.11 mm, *P* < 0.001) and less AE (by 0.22 ± 0.07 mm, *P* = 0.002) compared with the 6-MM group. A greater increase in total root mean square (RMS) of higher-order aberrations (HOAs), primary spherical aberration (SA) (${\mathrm{C}}_{4}^{0})$, and coma were also observed in the 5-MM group at all follow-up visits. The horizontal TZ diameter was significantly associated with changes in RMS HOAs, SA (RMS, primary and secondary SA), and RMS coma. After controlling for baseline parameters, RMS HOAs, RMS SA, RMS coma, and primary (${\mathrm{C}}_{4}^{0})$ and secondary (${\mathrm{C}}_{6}^{0})$ SA were significantly associated with AE.

**Conclusions:**

Ortho-k lenses with a smaller BOZD created a smaller horizontal TZ diameter and a significant increase in total HOAs, total SA, total coma, and primary SA and a decrease in secondary SA. Of these ocular aberrations, total HOAs, total SA, and primary SA were negatively correlated with AE over two years.

*Trial registration*: ClinicalTrial.gov, NCT03191942. Registered 19 June 2017, https://clinicaltrials.gov/ct2/show/NCT03191942.

**Supplementary Information:**

The online version contains supplementary material available at 10.1186/s40662-023-00344-3.

## Background

Myopia is a global health concern due to its increasing prevalence in recent decades [1], especially in Asian populations [2, 3]. There are various methods [4] to minimize myopia progression in children, of which atropine and orthokeratology (ortho-k) are most popular among practitioners [5].

Although ortho-k is an effective myopia control treatment, the mechanisms underlying how it slows eye growth in childhood remain unclear [6]. As ortho-k flattens the central cornea [the treatment zone [7, 8] (TZ)] and steepens the mid-peripheral cornea (the peripheral steepened zone [8]), the overall corneal shape is altered from a prolate to an oblate profile [6]. This results in decreased spherical refraction and increased corneal and ocular higher-order aberrations (HOAs) [9]. The increased root mean square (RMS) error for total HOAs after ortho-k is predominated by a positive shift in primary spherical aberration (SA) (due to corneal reshaping) and increased coma (typically due to lens decentration relative to the pupil) [10, 11]. Unlike lower-order aberrations (LOAs), HOAs cannot be corrected with conventional optical methods and consequently reduce retinal image quality, which typically manifests as a reduction in contrast sensitivity [12] and visual acuity [13], particularly under low illumination. These optical imperfections are thought to affect myopia progression in children undergoing ortho-k treatment.

Elevated corneal [14] and ocular [15] HOAs have been associated with reduced axial elongation (AE) in spectacle-wearing children, and similar significant negative associations have been reported in children wearing ortho-k lenses [16–18]. Recently, researchers have investigated novel ortho-k lenses designed to alter the corneal refractive profile by increasing the compression factor or reducing the back optic zone diameter (BOZD) [6]. It was hypothesized that these modified ortho-k lens designs can alter ocular aberrations (especially HOAs) and further retard AE compared with standard BOZD lenses.

Recent longitudinal studies have reported reduced AE in children using smaller BOZD ortho-k lenses, which create a significantly smaller TZ diameter compared with larger BOZD lenses [19, 20]. However, no studies have reported the longitudinal changes in ocular aberrations with smaller BOZD ortho-k lenses and their associations with AE. This study aimed to compare optical changes in children wearing 6-mm or 5-mm BOZD ortho-k lenses and to investigate their associations with AE over two years of follow-up.

## Methods

This study was a randomized clinical trial registered at ClinicalTrial.gov (NCT03191942) and approved by the Ethics Committee of the Hong Kong Polytechnic University (HSEARS20170118004). It was conducted following the tenets of the Declaration of Helsinki, according to the procedures described previously [19]. At least 24 subjects were required for each group to achieve 80% power to detect an 0.18-mm between group difference in AL (over two years) [19]. All subjects gave informed assent and parents and/or guardians provided informed consent prior to participation.

### Subjects

After fulfilling the inclusion criteria and passing lens handling training, subjects were randomly fitted with 6-mm (6-MM) or 5-mm (5-MM) BOZD ortho-k lenses in both eyes. All subjects were of Chinese ethnicity, aged 6 to < 11 years old, with myopia between − 4.00 to − 0.75 D, and anisometropia less than 1.00 D. Total astigmatism was required to be ≤ 2.50 D for with-the-rule axes (180º ± 30º) and ≤ 0.50 D for other axes. Subjects with other conditions were excluded from participating, including a history of myopia control treatment, strabismus or amblyopia, contraindications to contact lens/ortho-k wear, systemic/ocular conditions affecting refractive status, poor compliance with follow-ups and lens wear/handling, and poor visual/ocular response after lens modification.

Subjects were fitted with either spherical or toric BE Free Lenses (Precision Technology Services, Vancouver, B.C., Canada) with a series of lens diameters (10.2–11.2 mm) and a compression factor of 0.75 D for all lenses. Complimentary care solutions (Ophtecs Corporation, Tokyo, Japan) were provided for cleaning (rub and rinse), rinsing, disinfecting, and releasing bound lenses, respectively: O2 Daily Care Solution Pure, Cleadew saline, Cleadew GP, and Tiare W artificial tears. Subjects were instructed to wear ortho-k lenses for eight hours each night and attend regular aftercare visits.

### Examinations

Measurements were conducted prior to commencing ortho-k treatment (baseline visit) and every six months thereafter, within ± 2 h of the time of baseline visit, to reduce potential confounding effects of diurnal variations.

#### Corneal topography, pupil diameter, and treatment zone

Corneal topography was measured using an E300 Topographer (Version 6.1.2, Medmont International Pty. Ltd., Nunawading, VIC, Australia). Pupil diameter was estimated using the E300 under dark room lighting, but was considered a photopic pupil diameter measurement because of the internal equipment lighting (18.8 Lux) [20]. The TZ, enclosed by points with zero local paraxial power change, was determined by exporting raw tangential curvature data from the Medmont software which were fitted with a best-fit ellipse using a Python-based software developed previously [8]. The horizontal TZ diameter was selected to represent the TZ changes in the current study.

#### Aberrometry

Monochromatic (wavelength of 555 nm) ocular aberrations of the right eye were measured using a Shack-Hartmann aberrometer (COAS version 1.44.12, Wavefront Sciences Ltd., Albuquerque, NM, USA) in a dark room (five lux) with the left eye occluded. Participants viewed an external target through a Badal system, to correct their non-cycloplegic spherical equivalent refraction (SER) and ensure relaxed accommodation [21]. Measurements were repeated five times (each containing 25 frames) and averaged for analysis. The 125 measurements obtained at each visit were screened using customised software, excluding measurements with pupil size or RMS of total HOAs beyond the median of the sample, by 0.50 mm and 0.10 µm, respectively. The screened measurements were then fitted with a Zernike polynomial up to and including the 6th radial order and averaged.

Previous studies investigating HOAs have used a range of pupil diameters for analyses (4 mm [22], 5 mm [17], or 6 mm[16]). A 4-mm pupil diameter was used in the current study, as this is more likely to be the typical pupil diameter of children during daily activities (e.g., near work under photopic conditions) rather than a larger cycloplegic pupil diameter. The data was rescaled to a 4-mm pupil diameter using Schwiegerling’s technique [23], to avoid potential errors when extrapolating the Zernike polynomials to a larger pupil size. The RMS of total HOAs, individual Zernike coefficients of LOAs (2nd order) and HOAs (3th to 6th order), and RMS of SA (combination of ${C}_{4}^{0}$ and ${C}_{6}^{0}$) and coma (combination of ${C}_{3}^{-1}$, ${C}_{3}^{1}$, ${C}_{5}^{-1}$, and ${C}_{5}^{1}$) were calculated.

#### Subjective refraction and axial length

Two drops of 1% cyclopentolate were instilled with a 5-min interval in between, and full cycloplegia was achieved when there was no pupil reaction to light and less than 2.00 D accommodation (using an RAF ruler). Post-cycloplegic subjective refraction was assessed by the same practitioner using trial (ophthalmic) lenses. Axial length (AL) measurements (IOLMaster 500; Carl Zeiss Meditec AG, Jena, Germany) were performed after cycloplegia by a masked examiner (masked for AL measurements). Five measurements with a signal-to-noise ratio ≥ 5 and between-differences ≤ 0.02 mm were averaged for analysis.

### Statistical analysis

All statistical analyses were conducted using SPSS (ver. 26.0, IBM Corporation, Amonk, NY, USA). Only data from the right eyes were included in the analysis. Subjects with missing baseline HOAs were excluded. The normality of the data was investigated with the Shapiro-Wilk test. Unpaired t-test and Mann-Whitney test were performed to compare baseline measurements between subjects who completed and who dropped out from the study, and between the two groups, for normally or non-normally distributed data, respectively. Sex was compared using the Chi-squared test. Repeated measures ANOVA was used to examine changes in TZ diameter, AL, and various HOA and LOA metrics within subjects (time effect), between subjects (group effect), and their interaction (group by time interaction). Due to limited aberration data at the 18-month visit (6-MM group: n = 12; 5-MM group: n = 14) (interrupted data collection schedule due to social unrest and COVID-19), this visit was excluded from the repeated measures analysis. Post-hoc analysis was conducted using paired or unpaired t-tests with Bonferroni correction. Simple linear regression was used to evaluate the association between the horizontal TZ diameter and AE.

Horizontal TZ diameter at the 6-month visit was included as a fixed factor in all linear mixed models (LMMs). An initial LMM, using the pooled data (5-MM and 6-MM groups combined), was constructed to investigate the parameters (lens design, baseline age, sex, SER, AL, pupil diameter, and change in SER) associated with the horizontal TZ diameter. A series of LMMs (Models 1–5) were then used to investigate the association between baseline data (age, sex, SER, pupil diameter, AL, and corresponding HOAs or LOAs terms) and the horizontal TZ diameter with the changes in various ocular aberration terms. An LMM was applied to explore the effect of sex, TZ diameter at 6 months, and baseline data (pupil diameter, SER, best-corrected visual acuity, AL, RMS HOAs, and RMS LOAs) on AL over two years (Model 6), using pooled data and each group independently. Model 6 determined which baseline factors should be included in subsequent modelling (Models 7–11), which included aberration metrics. To explore the aberration terms associated with AL over two years, a series of LMMs, controlling for the significant baseline parameters found in Model 6, were used (Models 7–11), both with the pooled data and each group independently: Model 7 included the RMS HOAs; Model 8 analysed the RMS LOAs; Model 9 included RMS SA and RMS coma for analysis; Model 10 analysed primary (${\mathrm{C}}_{4}^{0}$) and secondary (${\mathrm{C}}_{6}^{0})$ SA; and Model 11 included each second order aberration term. All LMMs were constructed using a first-order autoregressive covariance and restricted maximum likelihood estimation, including individual subject’s slope and intercept as random effects and unstructured covariance matrix to control inter-subject variations. A backward stepwise approach was used, excluding the least significant factors to improve the fit of each model, based on the Akaike information criterion [24]. A *P* value of less than 0.05 was considered statistically significant.

## Results

Twenty-three subjects in 6-MM group and 22 subjects in 5-MM group completed the study, but only data from 22 and 21 subjects, respectively, were analysed as one subject in each group did not have baseline HOAs measurements. Baseline measurements of subjects who completed and dropped out from the study are shown in Table 1. More male subjects with longer initial AL, greater amount of initial myopia and SER dropped out in the 6-MM group, and relatively older subjects dropped out in the 5-MM group (*P* < 0.05). Reasons for dropouts included ocular health problems, unsatisfactory lens fit, and withdrawal of consent. No participants dropped out from the study due to rapid myopia progression. For subjects who completed the study, apart from astigmatism, there were no significant differences in baseline characteristics between the 6-MM and 5-MM groups. In spite of the dropouts, the study still had a power of 83% based on the between group comparison (AE) over two years using G-power analysis (Heinrich-Heine-Universität Düsseldorf, Düsseldorf, Germany; http://www.gpower.hhu.de/).

**Table 1:** Demographics and baseline data of subjects who completed and dropped out from the two-year study (median [range] or mean ± SD)

Parameters		6-MM			5-MM		
	*P’* (between groups)	Completed (n = 23)	Dropped out (n = 11)	*P*	Completed (n = 22)	Dropped out (n = 14)	*P*
Age (years)	0.40	9.32 ± 0.93	8.86 ± 1.09	0.22	9.03 ± 1.25	9.77 ± 0.67	0.027*
Male/female	0.29	6/17	7/4	0.035*	9/13	6/8	0.91
Axial length (mm)	0.07	24.24 ± 0.69	24.88 ± 0.56	0.012*	24.65 ± 0.79	24.37 ± 0.73	0.28
Myopia (D)	0.19	− 2.17 ± 0.81	− 3.05 ± 0.84	0.005*	− 2.47 ± 0.86	− 2.41 ± 0.73	0.84
Astigmatism (D)	0.043*	− 0.25 [− 0.50 to 0.00]	− 0.75 [− 1.25 to 0.00]	0.023*	− 0.50 [− 0.75 to 0.00]	− 0.50 [− 1.50 to 0.00]	0.76
SER (D)	0.11	− 2.31 ± 0.84	− 3.36 ± 0.98	0.002*	− 2.69 ± 0.89	− 2.67 ± 0.71	0.95
Pupil diameter (mm)	0.67	4.39 ± 0.84	4.58 ± 0.57	0.55	4.52 ± 0.90	4.47 ± 1.03	0.88

*SER* = spherical equivalent refraction; *n* = number of subjects

6-MM: using orthokeratology lenses with a back optic zone diameter of 6 mm; 5-MM: using orthokeratology lenses with a back optic zone diameter of 5 mm

*P*: probability value of unpaired-t or Mann-Whitney U tests for difference between subjects in each group who completed and dropped out from the study

*P’*: probability value of unpaired-t or Mann-Whitney U tests for difference between groups of subjects who completed the study

^*^Significantly different between groups or between subjects who completed or dropped out from the study

### Treatment zone diameter

One subject from the 5-MM group was excluded from the TZ analysis because of poor corneal response and TZ shape. The horizontal TZ diameter was significantly smaller in the 5-MM group compared with the 6-MM group, both at the 6-month (2.74 ± 0.29 vs. 3.68 ± 0.31 mm) and 24-month (2.69 ± 0.28 vs. 3.84 ± 0.39 mm) (all *P* < 0.001) visits. Similar results were found for the vertical TZ diameter at the 6-month (2.75 ± 0.25 vs. 3.53 ± 0.33 mm) and 24-month (2.65 ± 0.22 vs. 3.42 ± 0.35 mm) visits (all *P* < 0.001). Using a simple regression analysis with the pooled data, the horizontal TZ diameter at the 6-month visit was associated with AE after two years (standardized β = 0.431, adjusted R^2^ = 0.166, *P* = 0.004). Multivariate analysis using an LMM revealed that the horizontal TZ diameter was associated with lens design only (β =  − 0.95, *P* < 0.001, controlled for sex, baseline age, baseline SER, baseline AL, and change in SER).

### Ocular aberrations

No significant between-group differences were found in ocular aberrations at the baseline visit (*P* > 0.05, Table 2). Repeated measures analysis found a significant time (F = 57.16, *P* < 0.001), group (F = 55.18, *P* < 0.001), and group by time effect (F = 18.95, *P* < 0.001) for RMS of total HOAs over two years (Table 3). Significant time (F = 68.21, *P* < 0.001) and group (F = 5.68, *P* = 0.02) effects were observed for RMS of total LOAs, without group by time effect (*P* = 0.58).

**Table 2:** Baseline ocular aberration metrics of orthokeratology subjects in both groups (median [range] or mean ± SD)

Ocular aberration metrics (µm)	6-MM (n = 22)	5-MM (n = 21)	*P*
RMS of total lower-order aberrations	1.67 ± 0.53	1.99 ± 0.48	0.049*
${\mathrm{Defocus }(\mathrm{C}}_{2}^{0})$	1.65 ± 0.54	1.96 ± 0.47	0.052
RMS of total higher-order aberrations	0.10 ± 0.03	0.09 ± 0.03	0.319
RMS of spherical aberrations	0.03 ± 0.02	0.03 ± 0.02	0.605
Primary spherical aberration (${\mathrm{C}}_{4}^{0})$	0.03 ± 0.03	0.03 ± 0.02	0.874
Secondary spherical aberration (${\mathrm{C}}_{6}^{0})$	0.00 [0.00 to 0.00]	0.00 [− 0.02 to 0.00]	0.244
RMS of comatic aberrations	0.05 [0.02 to 0.13]	0.05 [0.01 to 0.14]	0.473
Primary vertical coma (${\mathrm{C}}_{3}^{-1})$	0.03 ± 0.06	0.03 ± 0.05	0.631
Primary horizontal coma (${\mathrm{C}}_{3}^{1}$)	0.00 ± 0.03	0.00 ± 0.03	0.586
Secondary vertical coma (${\mathrm{C}}_{5}^{-1})$	0.00 ± 0.00	0.00 ± 0.00	0.107
Secondary horizontal coma (${\mathrm{C}}_{5}^{1})$	0.00 ± 0.00	0.00 ± 0.00	0.513

*n* = number of subjects; *RMS* = root mean square

6-MM: using orthokeratology lenses with a back optic zone diameter of 6 mm; 5-MM: using orthokeratology lenses with a back optic zone diameter of 5 mm

*P*: probability value of unpaired-t or Mann-Whitney U tests for differences between groups

^*^Difference between groups were marginally significant

**Table 3:** Repeated measures analysis on the within-subjects and between-subjects effect on different ocular aberrations metrics

Ocular aberration metrics	*P*		
	Group effect	Time effect	Group by time effect
RMS of total lower-order aberrations	0.024*	< 0.001*	0.58
${\mathrm{Defocus }(\mathrm{C}}_{2}^{0})$	0.021*	< 0.001*	0.59
RMS of total higher-order aberrations	< 0.001*	< 0.001*	< 0.001*
RMS of spherical aberrations	< 0.001*	< 0.001*	< 0.001*
Primary spherical aberration (${\mathrm{C}}_{4}^{0})$	< 0.001*	< 0.001*	< 0.001*
Secondary spherical aberration (${\mathrm{C}}_{6}^{0})$	0.008*	0.39	0.11
RMS of comatic aberrations	0.004*	< 0.001*	0.007*
Primary vertical coma (${\mathrm{C}}_{3}^{-1})$	0.46	0.10	0.13
Primary horizontal coma (${\mathrm{C}}_{3}^{1}$)	0.07	0.14	0.36
Secondary vertical coma (${\mathrm{C}}_{5}^{-1})$	0.89	0.97	0.43
Secondary horizontal coma (${\mathrm{C}}_{5}^{1})$	0.65	0.43	0.67

^*^Significant time, group, or group by time interactions observed

Table 4 presents the ocular aberration metrics, which varied significantly between the two groups at each follow-up visit. A significant group effect, but not a time/group by time effect (*P* < 0.05), was observed for changes in HOAs (total HOA, total SA, total coma, and primary and secondary SA) starting from the 6-month visit, indicating that the between-group differences remained stable over the two-year study period. No group or time effect was found for the change of RMS LOA, defocus, or individual comatic aberration terms (${\mathrm{C}}_{3}^{-1}$, ${\mathrm{C}}_{3}^{1}$, ${\mathrm{C}}_{5}^{-1}$, and ${\mathrm{C}}_{5}^{1}$) after the 6-month visit.

**Table 4:** Ocular aberration metrics between groups at different follow-up visits (median [range] or mean ± SD)

	6-month			12-month			24-month		
Ocular aberrations (µm)	6-MM group (n = 21)	5-MM group (n = 18)	*P*	6-MM group (n = 18)	5-MM group (n = 18)	*P*	6-MM group (n = 19)	5-MM group (n = 20)	*P*
RMS LOAs	0.62 ± 0.28	0.91 ± 0.44	0.026	0.57 [0.27 to 2.04]	0.88 [0.28 to 1.82]	0.064	0.77 [0.29 to 1.36]	0.87 [0.36 to 2.22]	0.095
Defocus ${(\mathrm{C}}_{2}^{0})$	0.46 [0.19 to 1.13]	0.74 [0.07 to 1.68]	0.020	0.49 [0.17 to 2.01]	0.75 [0.18 to 1.81]	0.068	0.72 [0.11 to 1.25]	0.86 [0.31 to 2.20]	0.095
RMS HOAs	0.18 ± 0.08	0.35 ± 0.09	< 0.001*	0.18 [0.10 to 0.48]	0.37 [0.22 to 0.57]	< 0.001*	0.19 ± 0.05	0.39 ± 0.11	< 0.001*
RMS SA	0.07 [0.02 to 0.22]	0.24 [0.06 to 0.35]	< 0.001*	0.01 [− 0.02 to 0.03]	0.24 [0.08 to 0.37]	< 0.001*	0.09 ± 0.05	0.26 ± 0.11	< 0.001*
Primary SA (${\mathrm{C}}_{4}^{0})$	0.08 ± 0.08	0.23 ± 0.08	< 0.001*	0.07 ± 0.09	0.24 ± 0.09	< 0.001*	0.07 ± 0.08	0.25 ± 0.12	< 0.001*
Secondary SA (${\mathrm{C}}_{6}^{0})$	0.01 [− 0.01 to 0.03]	− 0.01 [− 0.03 to 0.05]	< 0.001*	0.06 [0.02 to 0.22]	− 0.01 [− 0.03 to 0.08]	0.068	0.01 ± 0.01	− 0.01 ± 0.02	0.004*
RMS coma	0.10 ± 0.05	0.20 ± 0.12	0.003*	0.10 [0.03 to 0.38]	0.20 [0.03 to 0.53]	0.051	0.13 ± 0.06	0.22 ± 0.12	0.006*

*RMS* = root-mean-square; *LOAs* = lower-order aberrations; *HOAs* = higher-order aberrations; *n* = number of subjects; RMS HOAs: from third to sixth orders (inclusive); RMS SA: ${\mathrm{C}}_{4}^{0}$ and ${\mathrm{C}}_{6}^{0}$ combined; RMS coma: ${\mathrm{C}}_{3}^{-1}$, ${\mathrm{C}}_{3}^{1}$, ${\mathrm{C}}_{5}^{-1}$, and ${\mathrm{C}}_{5}^{1}$ combined

6-MM: using orthokeratology lenses with a back optic zone diameter of 6 mm; 5-MM: using orthokeratology lenses with a back optic zone diameter of 5 mm

*P*: probability value of unpaired-t or Mann-Whitney U tests for differences between groups, after Bonferroni correction

^*^Significantly different between groups

Multivariate analysis showed that the change in RMS of total HOAs was associated with the horizontal TZ diameter (β =  − 0.15, *P* < 0.001, Additional file 1, Model 1), after controlling for baseline parameters. The TZ diameter was associated with the change in RMS of SA (β =  − 0.12, *P* < 0.001), RMS of coma (β =  − 0.05, *P* = 0.005), and primary ${(\mathrm{C}}_{4}^{0})$ (β =  − 0.15, *P* < 0.001) and secondary $({\mathrm{C}}_{6}^{0})$ SA (β = 0.02, *P* < 0.001) after taking baseline data into consideration. No significant associations were found between the TZ diameter and changes in RMS LOAs and defocus.

### Axial length/elongation

Significant time (F = 74.43, *P* < 0.001) and group effects (F = 16.96, *P* < 0.001) were observed for AE, with no group by time interaction, indicating that no further AE reduction was observed in the 5-MM group compared to 6-MM group after the 6-month visit. Post-hoc analysis showed significantly slower AE (*P* ≤ 0.002) in the 5-MM group at all follow-up visits. At the 24-month visit, on average, the 5-MM group showed 0.22 mm less AE compared with the 6-MM group (6-MM group: 0.35 ± 0.23 mm, n = 22; 5-MM group: 0.13 ± 0.19 mm, n = 21; *P* = 0.002).

Additional file 2 summarises the factors associated with AL over the two-year follow-up, using pooled data and each group independently. The RMS of total HOAs (Model 7) and total LOAs aberrations (Model 8) were only associated with AL in the 5-MM group and pooled data. No significant associations were found between total comatic aberrations (combination of ${\mathrm{C}}_{3}^{-1}$, ${\mathrm{C}}_{3}^{1}$, ${\mathrm{C}}_{5}^{-1}$, and ${\mathrm{C}}_{5}^{1}$) and AL, in either group or using pooled data (Model 9). Total SA (combination of ${\mathrm{C}}_{4}^{0}$ and ${\mathrm{C}}_{6}^{0}$) was significantly correlated with AL in the 5-MM group and pooled data, but not in the 6-MM group (Model 9). Primary SA (${\mathrm{C}}_{4}^{0}$) was significantly correlated with AE in the 5-MM group and with pooled data, but not in the 6-MM group (Model 10). For individual LOAs terms, defocus (${\mathrm{C}}_{2}^{0}$) was associated with AL in the 5-MM group only, and vertical astigmatism (${\mathrm{C}}_{2}^{2}$) was associated with AL in the 5-MM group and the pooled data (Model 11).

## Discussion

This study is the first to examine the effect of altering the BOZD of ortho-k lenses on ocular aberrations and the associations between the induced optical changes with eye growth in myopic children. The 5-mm ortho-k lenses resulted in a significantly smaller horizontal TZ diameter, greater RMS HOAs and RMS SA, and less AE over the study period. Multivariate analysis, controlling for other baseline factors, revealed significant associations between ocular aberrations such as increased SA, and reduced AE in ortho-k. Slower AE was associated with a greater increase in RMS HOAs and RMS SA, and a greater decrease in LOAs in the 5-MM group, while these associations were not observed in the 6-MM group. These results indicate that reducing the BOZD of ortho-k lenses alters the ocular aberration profile, which appears to contribute to a greater reduction in AE after two years, primarily due to the slower axial growth during the first six months of treatment.

Previous studies have reported a correlation between ocular aberrations and TZ diameter [25, 26]. Carracedo et al. [7] reported a significantly greater increase in corneal and total SA in the 5-mm BOZD group compared with the 6-mm BOZD group. The results from the current study are consistent with these previous studies but highlights the new association between increased HOAs (especially SA) and less AE in the 5-MM group.

In a retrospective analysis of patient clinical records, Pauné et al. [20] reported significantly less AE in subjects wearing ≤ 5 mm BOZD ortho-k lenses after one year of treatment compared with those using a BOZD > 5 mm (0.09 ± 0.12 *vs.* 0.15 ± 0.11 mm, *P* = 0.035). Similarly, the current study showed significantly less AE after one year for the 5-mm BOZD compared with 6-mm BOZD ortho-k lenses (0.04 ± 0.15 *vs.* 0.17 ± 0.13 mm, *P* = 0.001) [19]. Another study by Cheng et al. [27] compared AE in children wearing standard (spherical design) and test (with increased SA, + 0.175 µm across a 5-mm aperture) soft contact lenses over two years and found significantly less AE in the test group during the first year of treatment (6-month: 0.11 mm less; 12-month: 0.14 mm less). This magnitude of SA equates to approx. + 0.07 µm across a 4-mm pupil, which is similar to the levels observed in the 5-MM group in our study.

A significant group by time interaction was observed in the HOAs investigated, suggesting a greater increase in HOAs in the 5-MM group over two years. This is most likely because of the difference in TZ diameter between groups, which stabilized after the 6-month visit. Between-group difference in AE was also only observed during the first six months of lens wear, which suggests that the additional myopia control effect of smaller BOZD lenses (compared with standard lenses) occurs within the first six months, similar to the results reported by Tan et al.[28] who compared a combination therapy (atropine and ortho-k) with a monotherapy of ortho-k alone.

It should be highlighted that in this study, the subjects in 5-MM group exhibited greater change at the 6-month visit (presumably, after stabilization of treatment) in HOAs after ortho-k treatment compared with the subjects in 6-MM group. After commencing lens wear, HOAs measurements increased by 80%–167% in the 6-MM group and 289%–700% in the 5-MM group at the 6-month visit. Although affected by the pupil size (4 mm) used for analysis, it is meaningful that a reduction in TZ diameter of 1.14 mm can result in such dramatic changes in HOA.

Previous studies have found increased HOAs, SA, and coma in ortho-k subjects, and that AE was negatively associated with these ocular aberrations [16, 17]. Lau et al. [29] observed a greater increase in total RMS HOAs, total SA, and primary SA (${\mathrm{C}}_{4}^{0})$ in subjects wearing ortho-k lenses with an increased (1.75 D) compared to a conventional compression factor (0.75 D), during the first month of lens wear. A similar trend was found in the current study, with a greater increase in RMS of total HOAs, RMS of SA, and primary SA (${\mathrm{C}}_{4}^{0})$ in the 5-MM group. As anticipated, this confirms that increasing the compression factor or reducing the BOZD of ortho-k lenses result in a similar change to the HOA profile (i.e., an increase in primary SA (${\mathrm{C}}_{4}^{0})$). However, no such changes were reported in a one-year longitudinal study on adults comparing the same compression factors, with similar TZ diameters [30]. In their two-year longitudinal study, Lau et al. [31] reported slower AE in subjects wearing ortho-k lenses with increased compression factor. The TZ was smaller compared to those wearing lenses with a conventional compression factor, but the difference did not reach statistical significance. In a short-term study by Carracedo et al. [7], 12 subjects (18 eyes) were fitted with either 6-mm or 5-mm BOZD ortho-k lenses. The 5-mm BOZD lenses produced a significantly smaller TZ diameter (*P* < 0.05), increased 4th order, and total SA compared to the 6-mm design, which adversely affected contrast sensitivity, but not low contrast visual acuity.

After adjusting for baseline parameters, in the pooled data, RMS LOAs, RMS HOAs, RMS SA, and primary SA were significantly associated with AE. However, no such association was found for secondary SA, RMS coma, and individual comatic terms. Similar results were observed in the 5-MM group only. Compared with previous studies, the different results obtained may be due to the relatively smaller pupil diameter used and a smaller sample size. Lau, et al. [16] analysed data from 103 subjects using a 5-mm pupil. Hiraoka et al. [17] used the same pupil diameter as in the current study, but with a larger sample size of 55 subjects. Vincent et al. [21] compared changes in subjects wearing ortho-k lenses alone with those using a combined ortho-k and 0.01% atropine therapy. They found significant negative correlations between AE and total RMS HOAs and primary SA (${\mathrm{C}}_{4}^{0})$ in both pooled data and in combined therapy group for photopic pupil diameters, but no such association in their ortho-k alone group.

A limitation of this study is the relatively high drop-out rate (6-MM group: 32%; 5-MM group: 39%) and missing data of several subjects at the 18-month visit. Despite the reduced sample size, the results show that a 1-μm increase in total SA or primary SA was associated with 0.23 mm less AE after two years, controlling for other baseline factors. In addition, as mentioned earlier, the study still achieved an 83% power despite the dropouts.

## Conclusions

The 5-mm BOZD ortho-k lenses created a smaller horizontal TZ diameter compared with 6-mm lenses, resulting in greater increases in total HOAs, total SA, total coma, and primary SA, and a significantly greater decrease in secondary SA for a 4-mm photopic pupil diameter. Total HOAs, total SA, and primary SA, taking into account baseline parameters, were negatively correlated with AE over two years. Reducing the BOZD of ortho-k lenses is a viable method to enhance the reduction in AE and appears to be associated with changes in the HOA profile.

## Supplementary Information

**Additional file 1**. Statistically significant fixed effects and estimates (β) of influences on changes of ocular aberrations using pooled data (independent valuables included in each model; baseline age, SER, sex, pupil diameter, corresponding HOAs or LOAs terms, and horizontal TZ diameter) (previously published in PhD thesis).

**Additional file 2**. Statistically significant fixed effects and estimates (β) of influences on axial length using pooled data, and data from the 5-MM and 6-MM groups (previously published in PhD thesis).

## Data Availability

This study was supported by PolyU Research Resident Theme. When applying for ethics application (back in 2017), it was agreed that data would be kept confidential and only be accessible to team members and hence cannot be shared.

## Abbreviations

AE: Axial elongation
AL: Axial length
BOZD: Back optic zone diameter
HOAs: Higher-order aberrations
LMM: Linear mixed models
LOAs: Lower-order aberrations
Ortho-k: Orthokeratology
RMS: Root mean square
SA: Spherical aberration
SER: Spherical equivalent refraction
TZ: Treatment zone

## Declarations

The results of this study have been previously presented at the International Myopia Conference 2022. The results have been reported in the PhD thesis entitled “Variation of orthokeratology lens treatment zone (VOLTZ) study”.
