# Supplementary material for: Optical changes and association with axial elongation in children wearing orthokeratology lenses of different back optic zone diameter
Source: Eye Vis (Lond). 2023 Jul 1;10:25. doi: 10.1186/s40662-023-00344-3 (PMC10314406; doi:10.1186/s40662-023-00344-3)
Supplement: Supplementary file 1 — Additional file 1. Statistically significant fixed effects and estimates (β) of influences on changes of ocular aberrations using pooled data (independent valuables included in each model; baseline age, SER, sex, pupil diameter, corresponding HOAs or LOAs terms, and horizontal TZ diameter) (previously published in PhD thesis). [file 40662_2023_344_MOESM1_ESM.docx]

**Additional file 1.** Statistically significant fixed effects and estimates (β) of influences on changes of ocular aberrations using pooled data (independent valuables included in each model; baseline age, SER, sex, pupil diameter, corresponding HOAs or LOAs terms, and horizontal TZ diameter) (previously published in PhD thesis).

| **Parameters** | **β** | ***P*** |
| --- | --- | --- |
| Model 1: parameters affecting the change in RMS of HOAs |  |  |
| Intercept | 1.07 | 0.024 |
| 6-month horizontal treatment zone diameter | −0.19 | < 0.001* |
| Baseline RMS HOAs | −0.74 | 0.042 |
| Model 2: parameters affecting the change in RMS of SA |  |  |
| Intercept | 0.90 | 0.026 |
| 6-month horizontal treatment zone diameter | −0.16 | < 0.001* |
| Model 3: parameters affecting the change in RMS of Coma |  |  |
| Intercept | 0.31 | 0.577 |
| 6-month horizontal treatment zone diameter | −0.09 | < 0.001* |
| Model 4: parameters affecting the change in primary SA |  |  |
| Intercept | 0.83 | 0.047 |
| 6-month horizontal treatment zone diameter | −0.17 | < 0.001* |
| Model 5: parameters affecting the change in secondary SA |  |  |
| Intercept | −0.10 | 0.182 |
| Baseline SER | −0.01 | < 0.001* |
| 6-month horizontal treatment zone diameter | 0.02 | < 0.001* |

*RMS* = root mean square; *HOAs* = higher-order aberrations; *SA* = spherical aberration; *SER* = spherical equivalent refraction; RMS HOAs: from third to sixth orders (inclusive); RMS SA: $C_{4}^{0}$ and $C_{6}^{0}$ combined; RMS coma: $C_{3}^{-1}$, $C_{3}^{1}$, $C_{5}^{-1}$, and $C_{5}^{1}$ combined; *P*: probability value of multivariate association using linear mixed models; *: significant fixed effect found on changes of ocular aberrations
