# Supplementary material for: Optical changes and association with axial elongation in children wearing orthokeratology lenses of different back optic zone diameter
Source: Eye Vis (Lond). 2023 Jul 1;10:25. doi: 10.1186/s40662-023-00344-3 (PMC10314406; doi:10.1186/s40662-023-00344-3)
Supplement: Supplementary file 2 — Additional file 2. Statistically significant fixed effects and estimates (β) of influences on axial length using pooled data, and data from the 5-MM and 6-MM groups (previously published in PhD thesis). [file 40662_2023_344_MOESM2_ESM.docx]

**Additional file 2.** Statistically significant fixed effects and estimates (β) of influences on axial length using pooled data, and data from the 5-MM and 6-MM groups (previously published in PhD thesis).

| **Parameters** | **All subjects (n=43)** | | **6-MM (n=22)** | | **5-MM (n=21)** | |
| --- | --- | --- | --- | --- | --- | --- |
|  | **β** | ***P*** | **β** | ***P*** | **β** | ***P*** |
| Model 6: baseline parameters and 6-month treatment zone diameter | | | | | | |
| Intercept | −2.21 | 0.080 | −2.03 | 0.474 | −1.49 | 0.366 |
| Baseline axial length | 1.09 | < 0.001* | 1.12 | < 0.001* | 1.04 | < 0.001* |
| 6-month treatment zone diameter | 0.12 | 0.007* | 0.02 | 0.926 | 0.14 | 0.236 |
| Time | 0.06 | < 0.001* | 0.08 | < 0.001* | 0.03 | < 0.001* |
| Model 7: RMS of HOAs | | | | | | |
| Intercept | −0.51 | 0.503 | −0.66 | 0.588 | −0.32 | 0.719 |
| Baseline axial length | 1.01 | < 0.001* | 1.04 | < 0.001* | 0.99 | < 0.001* |
| 6-month treatment zone diameter | 0.07 | 0.071 | −0.09 | 0.397 | 0.16 | 0.069 |
| RMS HOAs | −0.22 | < 0.001* | 0.03 | 0.805 | −0.22 | 0.001* |
| Time | 0.06 | < 0.001* | 0.08 | < 0.001* | 0.05 | < 0.001* |
| Model 8: RMS of LOAs | | | | | | |
| Intercept | −0.59 | 0.457 | −0.66 | 0.585 | −0.32 | 0.718 |
| Baseline axial length | 1.01 | < 0.001* | 1.04 | < 0.001* | 0.98 | < 0.001* |
| 6-month treatment zone diameter | 0.10 | 0.017* | −0.08 | 0.416 | 0.22 | 0.018***** |
| RMS LOAs | 0.03 | 0.025* | −0.01 | 0.631 | 0.07 | < 0.001* |
| Time | 0.06 | < 0.001* | 0.08 | < 0.001* | 0.05 | < 0.001* |
| Model 9: RMS of SA and RMS coma | | | | | | |
| Intercept | −0.52 | 0.501 | −0.65 | 0.592 | −0.32 | 0.724 |
| Baseline axial length | 1.01 | < 0.001* | 1.04 | < 0.001* | 0.99 | < 0.001* |
| 6-month treatment zone diameter | 0.07 | 0.080 | −0.08 | 0.417 | 0.16 | 0.084 |
| RMS SA | −0.24 | 0.004* | 0.22 | 0.237 | −0.27 | 0.001* |
| Time | 0.07 | < 0.001* | 0.08 | < 0.001* | 0.05 | < 0.001* |
| Model 10: spherical Zernike terms ($C_{4}^{0}$and $C_{6}^{0}$) | | | | | | |
| Intercept | −0.58 | 0.456 | −0.69 | 0.571 | −0.41 | 0.655 |
| Baseline axial length | 1.01 | < 0.001* | 1.04 | < 0.001* | 1.00 | < 0.001* |
| 6-month treatment zone diameter | 0.08 | 0.065 | −0.08 | 0.447 | 0.16 | 0.076 |
| $C_{4}^{0}$ | −0.23 | 0.003* | 0.21 | 0.206 | −0.31 | < 0.001* |
| Time | 0.07 | < 0.001* | 0.08 | < 0.001* | 0.05 | < 0.001* |
| Model 11: LOAs Zernike terms | | | | | | |
| Intercept | −0.64 | 0.428 | −0.68 | 0.574 | −0.44 | 0.625 |
| Baseline axial length | 1.01 | < 0.001* | 1.04 | < 0.001* | 0.99 | < 0.001* |
| 6-month treatment zone diameter | 0.09 | 0.026* | −0.09 | 0.378 | 0.22 | 0.020* |
| $C_{2}^{0}$ | 0.02 | 0.119 | 0.00 | 0.875 | 0.05 | < 0.001* |
| $C_{2}^{2}$ | −0.09 | 0.039* | 0.04 | 0.664 | −0.09 | 0.024* |
| Time | 0.07 | < 0.001* | 0.08 | < 0.001* | 0.05 | < 0.001* |

Model 6 explores the baseline data that are significantly associated with axial length, these significant baseline values were included in Model 7–11 as independent values, together with other ocular aberrations metrics indicated in the table.

*RMS* = root mean square; *HOAs* = higher-order aberrations; *LOAs* = lower-order aberrations; *SA* = spherical aberration; RMS HOAs: from third to sixth orders (inclusive); RMS SA: $C_{4}^{0}$ and $C_{6}^{0}$ combined; RMS coma: $C_{3}^{-1}$, $C_{3}^{1}$, $C_{5}^{-1}$, and $C_{5}^{1}$ combined; 6-MM: using orthokeratology lenses of BOZD 6 mm; 5-MM: using orthokeratology lenses of BOZD 5 mm

*P*: probability value of multivariate association using linear mixed models; *: significant fixed effect found on axial length
